# Internal Hernia Through a Mesenteric Defect Presenting As Midgut Volvulus: A Rare and Intriguing Coexistence

**DOI:** 10.7759/cureus.85426

**Published:** 2025-06-05

**Authors:** Anshita Shrivastava, Summi Karn, Amulya Reddy, Karamveer Singh, Navin Kumar

**Affiliations:** 1 Department of General Surgery, All India Institute of Medical Sciences, Rishikesh, Rishikesh, IND

**Keywords:** bowel obstruction, emergency abdominal surgery, internal hernia, mesenteric defect, midgut volvulus

## Abstract

Internal hernias through mesenteric defects are a rare cause of bowel obstruction. Early recognition and intervention are crucial for preventing bowel ischemia and subsequent complications. We report a case of a 64-year-old female who presented with acute intestinal obstruction secondary to an internal hernia associated with volvulus. She had a previous surgical history of exploratory laparotomy 25 years back. This case illustrates the diagnostic challenges of internal hernias and emphasizes the importance of considering this entity in patients with previous abdominal surgery presenting with bowel obstruction. A CT scan helps in prompt diagnosis, and early surgery is required for the management. The concurrent finding of midgut volvulus adds to the uniqueness of this presentation.

## Introduction

Internal hernias are complex and life-threatening conditions where intestinal segments protrude through defects in the peritoneal ligament, mesentery, or omentum, without traversing the fascial planes and becoming entrapped within another compartment of an otherwise intact abdominal cavity [1]. These apertures or openings may occur in anatomically normal locations, such as the foramen of Winslow, or paranormal sites, including the paraduodenal, ileocecal, or supravesical fossa. Alternatively, they may be present through abnormal apertures, such as transomental defects. Internal abdominal hernias are rare in the adult population, with an incidence rate of only 0.2-0.9% [2].

The diagnosis of internal hernias is a challenge for both clinicians and radiologists. Patients often present with varied and nonspecific symptoms, primarily manifesting as small bowel obstruction [1]. The intermittent nature of symptoms, combined with the subtle clinical findings, frequently leads to missed or delayed diagnosis even after multiple healthcare encounters. If not promptly recognized and managed, the risk of strangulation is high [1,2]. The complexity of diagnosis, coupled with the potential for severe complications, makes internal hernias an important consideration in the differential diagnosis of acute abdomen, particularly in cases of unexplained bowel obstruction.

We present a case of acute intestinal obstruction due to internal herniation with midgut volvulus.

## Case presentation

**Figure 1:**
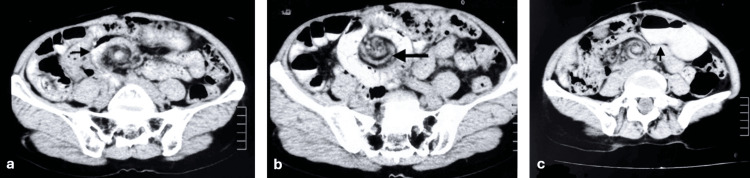
CECT abdomen showing (a) a swirl sign suggestive of midgut volvulus, (b) twisting of the mesenteric vessels, and (c) dilated proximal bowel loops with air-fluid levels CECT: contrast-enhanced computed tomography

A 64-year-old woman presented to our emergency department with features of acute intestinal obstruction on the background of vague abdominal pain for over a month. The patient had a past surgical history of exploratory laparotomy performed 25 years ago, supposedly for gangrenous small bowel requiring resection and anastomosis, though detailed operative records were unavailable. She had no known comorbidities. On physical examination, the patient appeared restless and distressed. Vital signs revealed tachycardia (110 beats per minute) with normal blood pressure and oxygen saturation. Abdominal examination demonstrated a non-distended, scaphoid abdomen with a right lower paramedian scar from previous surgery, without any tenderness. Hernial orifices were normal. Bowel sounds were exaggerated on auscultation. Digital rectal examination was unremarkable. Blood investigations, including complete blood count and lactate, were within normal limits. Initial resuscitation was initiated in the surgical high dependency unit. The contrast-enhanced abdominal CT revealed midgut volvulus with a positive swirl sign, with dilatation of proximal bowel loops and twisting of mesenteric features that were suggestive of midgut volvulus (Figure 1).

**Figure 2:**
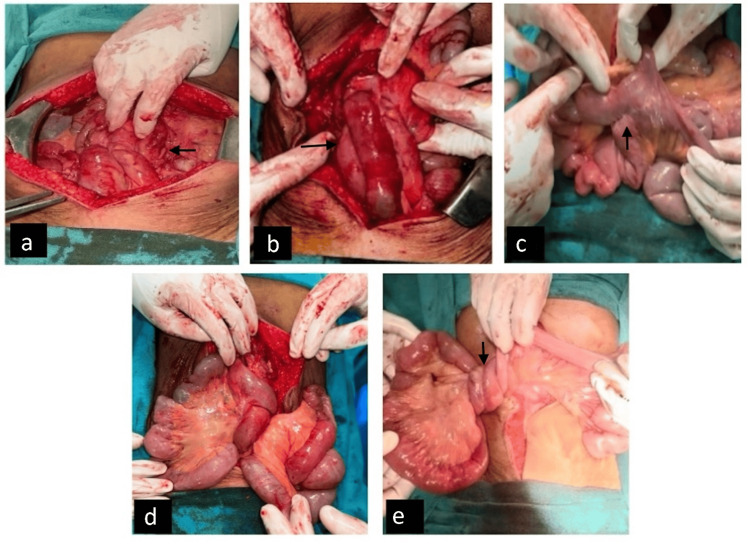
Intraoperative images showing (a) clumped bowel loops, (b) small bowel loops twisting round each other, presenting as midgut volvulus, (c) preexisting jejunoileal anastomosis, and (d, e) intestinal loops herniating through the mesenteric defect

The patient was admitted, and initial resuscitation with intravenous fluids, urinary catheterization, and monitoring of fluid balance was done. Following adequate resuscitation, emergency exploratory laparotomy was done. Intraoperatively, there was extensive small bowel volvulus, characterized by twisted jejunal and ileal loops around their mesenteric axis and clumped loops due to multiple inter-mesenteric and inter-bowel adhesions. A preexisting jejunoileal anastomosis was identified approximately 100 cm distal to the duodenojejunal junction. Jejunal and ileal loops were found herniating through the mesenteric defect, probably resulting from inadequate closure of the mesentery during the patient's prior surgery 25 years ago (Figure 2). The volvulus was carefully de-rotated, and the entire bowel was inspected for ischemia or compromised viability. Bowel resection was not required as no ischemic segments were noted, and normal peristalsis was present. The mesenteric defect was closed using interrupted, non-absorbable sutures to prevent its recurrence. The patient's postoperative course was unremarkable. She demonstrated appropriate recovery milestones, tolerated an oral diet by the third postoperative day, and was discharged on the fifth postoperative day with normal bowel function and adequate pain control.

## Discussion

Internal hernias are a rare cause of small bowel obstruction, with transmesenteric hernias forming a particularly uncommon subset [2]. Ghahremani classified internal hernia into congenital and acquired forms, with the latter being more prevalent in adults [3]. The causes of mesenteric defect formation remain unclear. Several hypotheses have been proposed for congenital cases, including dorsal mesenteric regression, developmental expansion of a poorly vascularized region, rapid lengthening of a segment of the mesentery, or the colon compressing the mesentery during fetal midgut herniation [2]. In 1885, Treves' theory described the mesentery of the terminal ileum as the weakest area [4]. The transmesenteric hernias account for only 5% to 10% of congenital internal hernias [5]. The acquired cases are frequently attributed to prior abdominal surgeries, postoperative adhesions, and various bypass procedures, particularly hepatobiliary and bariatric surgeries [5]. A transmesenteric hernia often occurs after previous surgery where the mesentery has been incised but not closed following gastrointestinal reconstruction [2]. As seen in our case, previous abdominal surgery involving bowel handling and manipulation appears to be the most probable cause of the mesenteric defect, which subsequently led to internal herniation and eventual volvulus of the midgut.

Congenital hernias may present with varying features, ranging from intermittent abdominal pain, the most common symptom, to acute intestinal obstruction, which may even be fatal [4,5]. Clinical manifestations are typically nonspecific, encompassing vague abdominal discomfort, abdominal distension, vomiting, and recurrent intestinal obstruction [6]. Preoperative diagnosis is challenging due to the broad range of possible presentations [7]. Symptoms such as obstructive jaundice, biliary colic, secondary pancreatitis, and sometimes, a palpable abdominal mass may also occur [1]. Internal herniation of abdominal organs, such as the spleen, is at constant risk of trauma, torsion, or infarction [8]. Approximately 66% of cases eventually progress to acute obstruction or strangulation, with closed-loop obstruction carrying a particularly high risk of bowel perforation [1]. In our patient, the classical features of acute intestinal obstruction, that is, acute abdominal pain, vomiting, and non-passage of flatus and feces, were present, along with complaints of vague abdominal pain for over a month. This depicts an acute-on-subacute presentation of recurrent subacute episodes of internal herniation of the small bowel through the mesenteric defect, which eventually led to volvulus of the jejunal and proximal ileal loops, presenting acutely as small intestinal obstruction.

While ultrasonography can aid in diagnosis through the identification of the clockwise whirlpool sign and abnormal mesenteric vessel relationships, as well as an inverted superior mesenteric artery (SMA)/superior mesenteric vein (SMV) relationship and a dilated duodenum proximal to the obstruction, abdominal CT scans with contrast remain the cornerstone in preoperative planning and surgical management [6-8]. Key findings in CT scans that can guide preoperative diagnosis of internal hernias include (a) recognition of abnormally positioned bowel clusters forming a saclike structure, (b) observation of a saclike structure or cluster of dilated small bowel loops at an abnormal anatomic location in the presence of small bowel obstruction, and (c) identification of engorged, stretched, and displaced mesenteric vessels that converge at the hernial orifice [6]. The pathognomonic CT findings of midgut volvulus are the characteristic "whirlpool sign" of mesentery and SMV wrapping around the SMA, along with the "beak" appearance of the obstructed proximal bowel [9]. The reported sensitivity of the whirlpool sign for small intestine volvulus is 64%, with a positive predictive value of 21% [10]. In our case, while CT demonstrated features of small bowel obstruction and the whirlpool sign, the internal hernia as the underlying cause was not definitively apparent, highlighting the diagnostic complexities of this condition. A high index of suspicion and clinical awareness are essential for the timely diagnosis and management of rare surgical emergencies, especially in adults with a previous history of abdominal surgeries. Delay in diagnosis can lead to bowel ischemia, necrosis, and perforation, significantly increasing morbidity and mortality.

Surgical exploration remains the gold standard for both diagnosis and treatment. Immediate surgical exploration following appropriate resuscitation is crucial, as delay can result in bowel ischemia and subsequent necrosis. The surgical approach follows a systematic sequence: reduction of the hernia, assessment of bowel viability, resection of non-viable segments if necessary, and definitive repair of the mesenteric defect [11,12]. The surgical strategy must be tailored to individual patient factors, including hemodynamic stability, extent of bowel compromise, and anatomical considerations. If resection is performed, stoma formation is usually required. Extensive bowel necrosis, necessitating resection, can lead to short bowel syndrome, thereby increasing the mortality rate [13]. Postoperative mortality and morbidity range from 2% to 30% and from 12% to 14%, respectively, depending on the timing of presentation and the promptness of surgical intervention [14]. Closure of mesenteric defects during surgery is essential to prevent recurrence. Some surgeons advocate for additional procedures, such as duodenal or cecal pexy. However, the long-term benefits of these adjunctive measures in preventing recurrence remain debatable, with a reported rate of around 14% [15,16]. The recurrence risk is seen to be increased in other mesenteric gaps, which should also be carefully examined during the surgery and closed [16]. Some studies advocate the use of mesh to reinforce the defect and reduce the risk of recurrence. However, mesh placement must be considered cautiously, especially in contaminated fields, due to the risk of infection [17].

While closure of mesenteric defects reduces the risk of internal herniation, it is not without complications. Moreover, the size of the mesenteric defect plays a crucial role in decision-making. Large defects may allow bowel loops to pass without significant risk of incarceration or strangulation, and thus closure may not be necessary. Conversely, a smaller defect poses a higher risk for complications, and closure is often considered. However, it is important to note that closing mesenteric defects can lead to morbidity, such as bleeding from the mesentery, which must be weighed against the potential benefits [18].

Internal hernia through mesenteric defects is a rare but critical differential diagnosis in acute intestinal obstruction. This case is particularly noteworthy for the concurrent presentation of internal hernia and midgut volvulus, a rare combination that presents unique diagnostic and therapeutic challenges. While internal hernias alone may be encountered, their association with midgut volvulus is rare, a seldom-seen phenomenon in surgical practice. Through prompt diagnosis and timely intervention, we successfully preserved the bowel, averting the inevitable ischemia or necrosis associated with either volvulus or hernia over time. The scarcity of published reports describing this dual pathology underscores the significance of our case in expanding the existing knowledge base. It serves as a valuable reference for surgeons encountering similar complex presentations, highlighting the importance of considering these rare entities in the differential diagnosis of acute bowel obstruction, particularly in patients with a history of previous abdominal surgery. Furthermore, this case reinforces the fundamental surgical principle of meticulous mesenteric closure during intestinal surgery to prevent small bowel obstruction and the need for reoperation, as concluded in multiple other randomized controlled trials [19,20].

## Conclusions

Internal hernias with concurrent midgut volvulus represent a rare but potentially devastating cause of acute bowel obstruction, particularly in patients with previous abdominal surgery. While the clinical and radiological features were evident in our case, these conditions can often present with variable symptoms and subtle imaging findings, making diagnosis challenging in many instances. This emphasizes the importance of maintaining a high index of clinical suspicion. Prompt surgical intervention remains crucial for optimal outcomes. Our case highlights several key principles: the importance of meticulous mesenteric closure during initial surgery, the value of early surgical exploration in suspected cases, and the significance of tailoring the surgical approach based on individual patient factors. This experience contributes to the limited literature on this rare dual pathology, offering valuable insights for informed surgical decision-making.
